# Supplementary material for: Extraction of Bioactive Compounds for Antioxidant, Antimicrobial, and Antidiabetic Applications
Source: Molecules. 2022 Sep 13;27(18):5935. doi: 10.3390/molecules27185935 (PMC9503716; doi:10.3390/molecules27185935)
Supplement: Supplementary file 1 [file molecules-27-05935-s001.zip › molecules-1889455-supplementary.pdf]

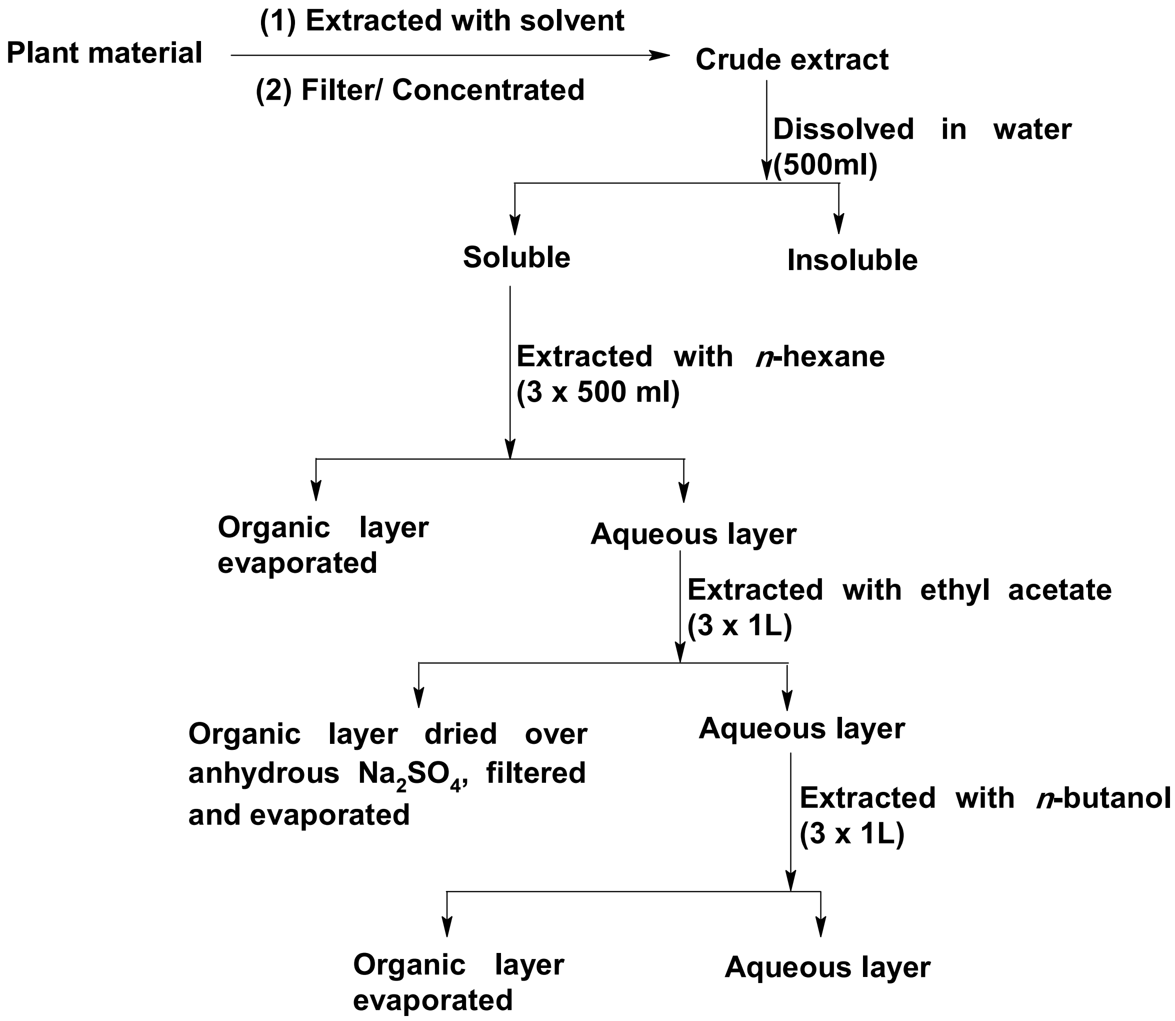

SS1. Scheme followed for the extraction of *A. arvensis,* & *A. americana.*

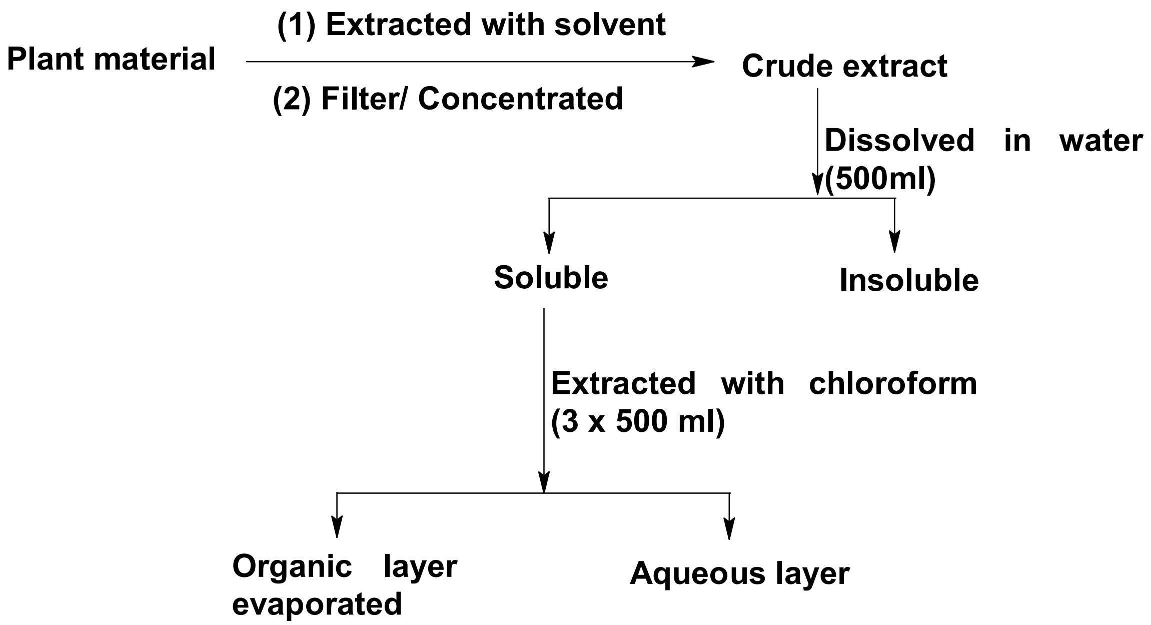

SS2. The scheme followed for the extraction of *C. colocynthis, S. nigrum, S. surattense,* and *C. procera*.

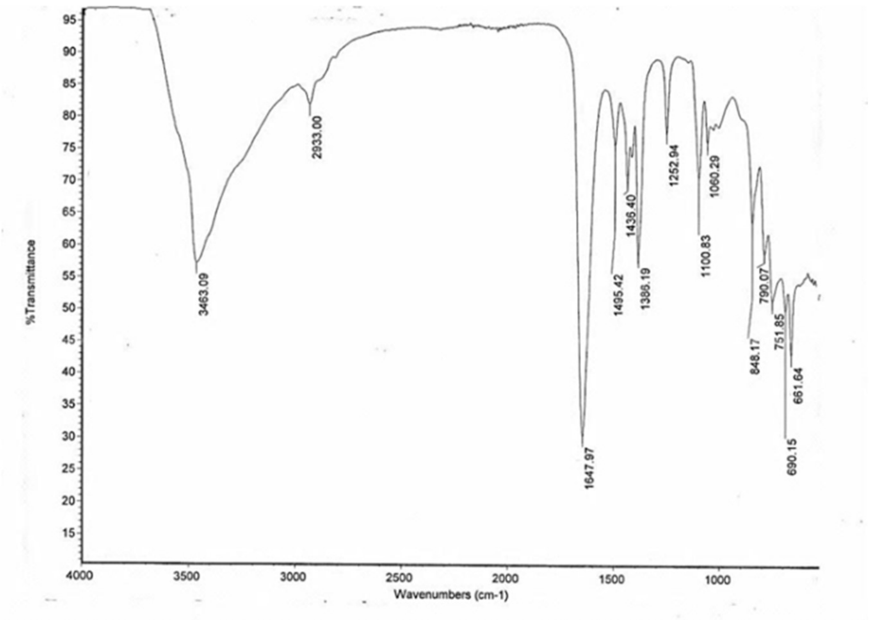

S 1. FT-IR spectrum of *S. surrattense* chloroform fraction of leaves.

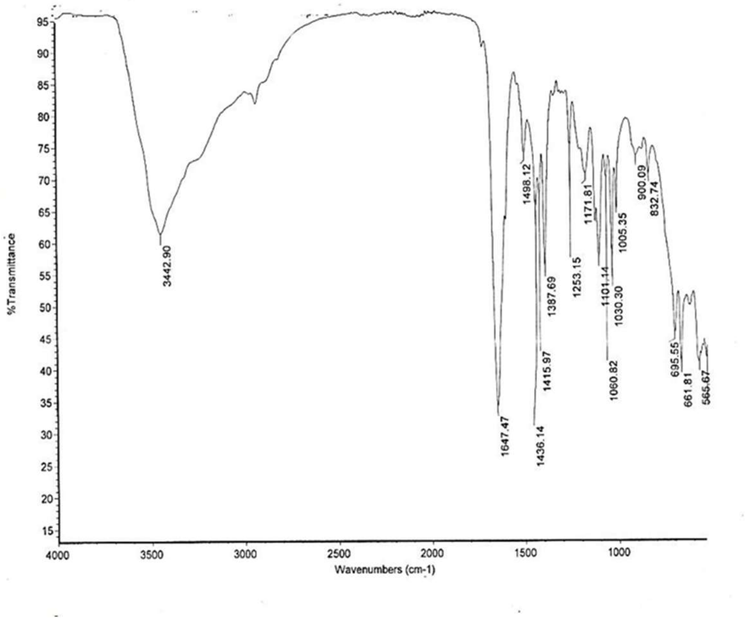

S 2. FT-IR spectrum of *S. surrattense* chloroform fraction of fruit extract.

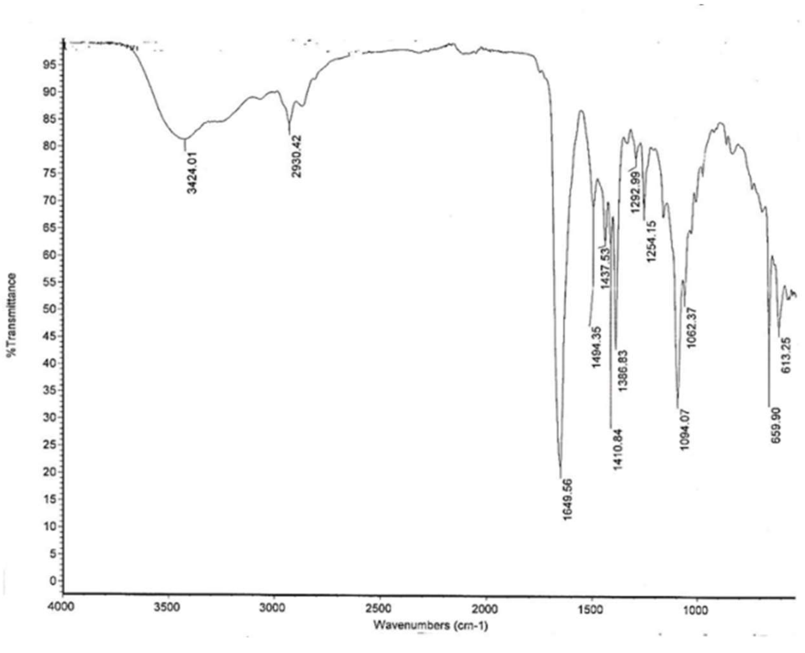

S 3. FT-IR spectrum of *S. surrattense* aqueous fraction of leave extract.

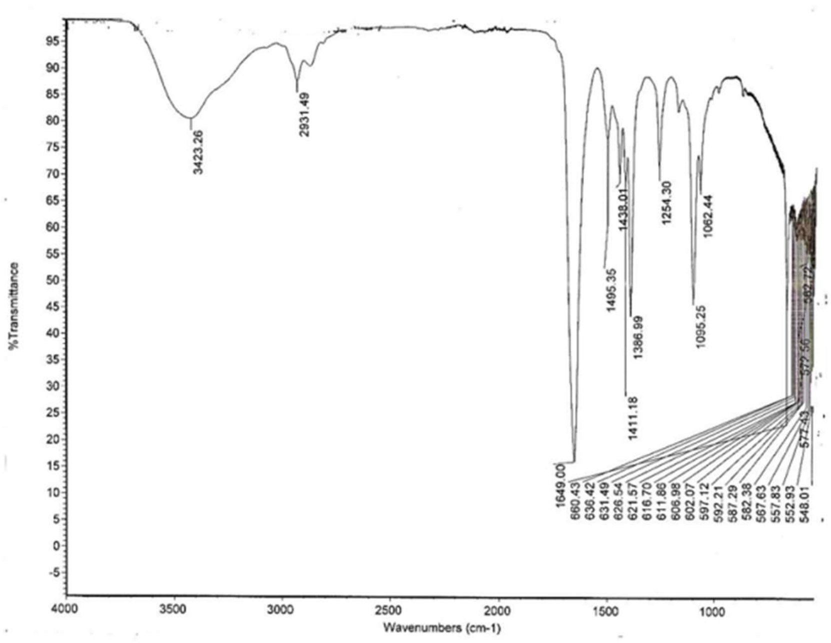

S 4. FT-IR spectrum of *S. surrattense* aqueous fraction of fruit extract.

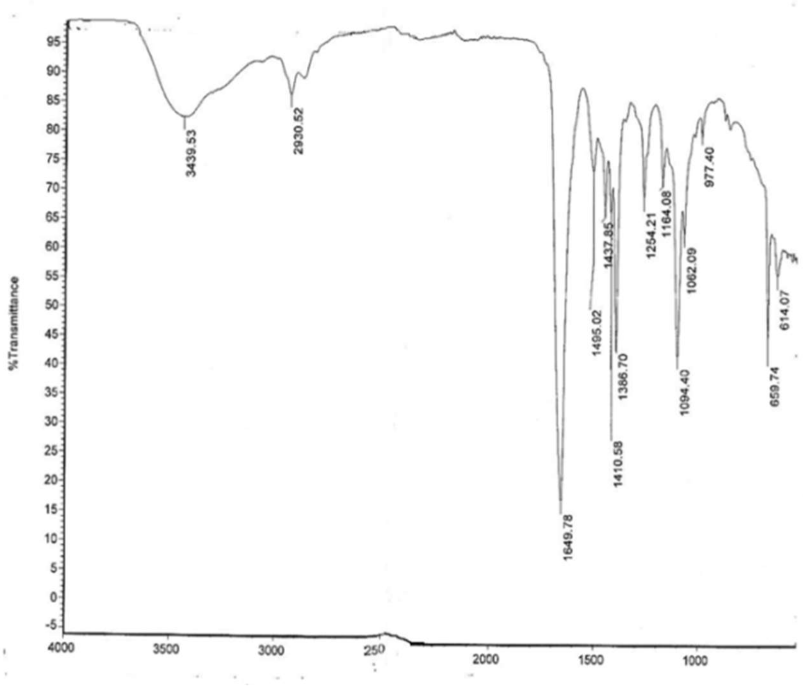

S 5. FT-IR spectrum of aqueous fraction of *C. procera.*

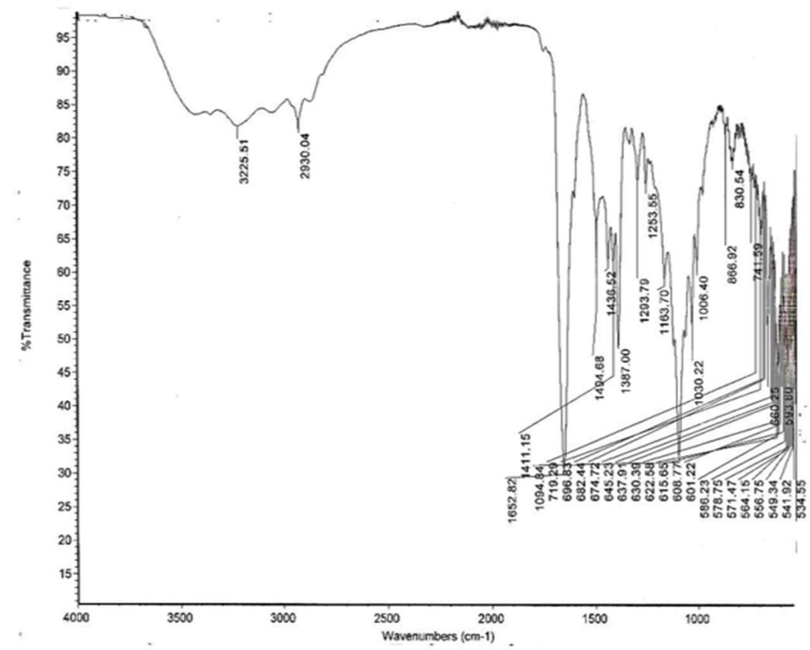

S 6. FT-IR spectrum of aqueous fraction of *C. colocynthis.*

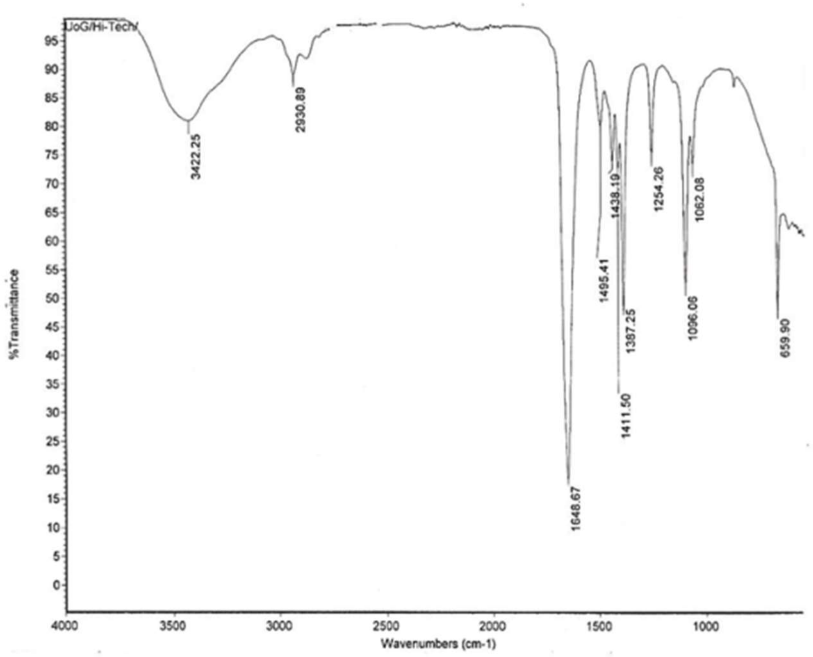

S 7. FT-IR spectrum of butanol fraction of *A. arvensis.*

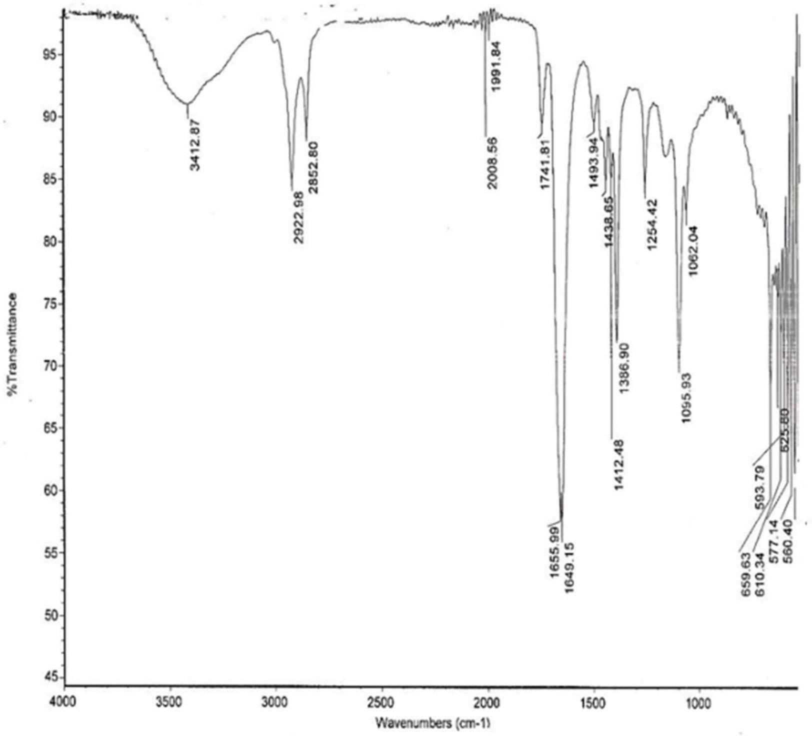

S 8. FT-IR spectrum of n-Hexane fraction of *A. arvensis.*

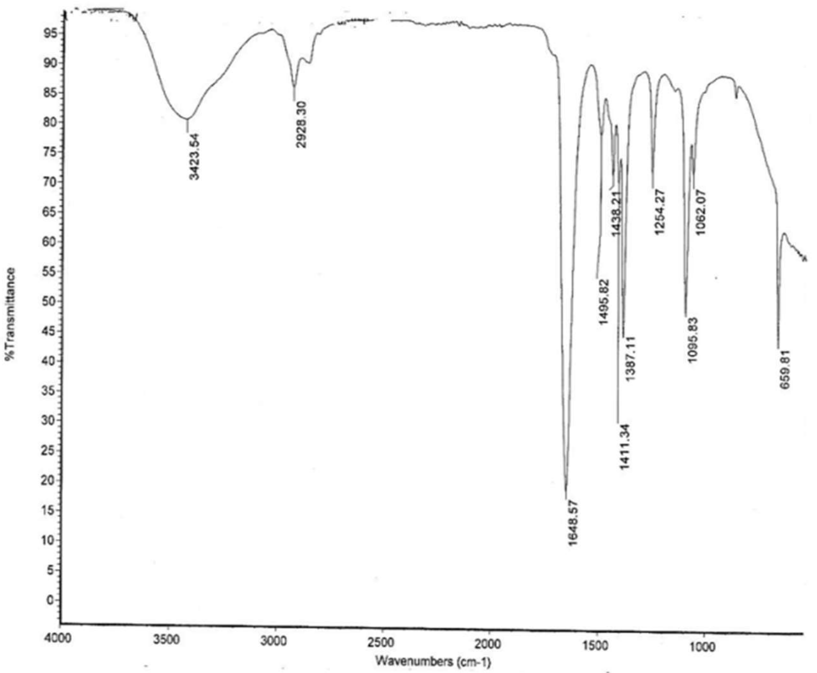

S 9. FT-IR spectrum of ethyl acetate fraction of *A. americana.*

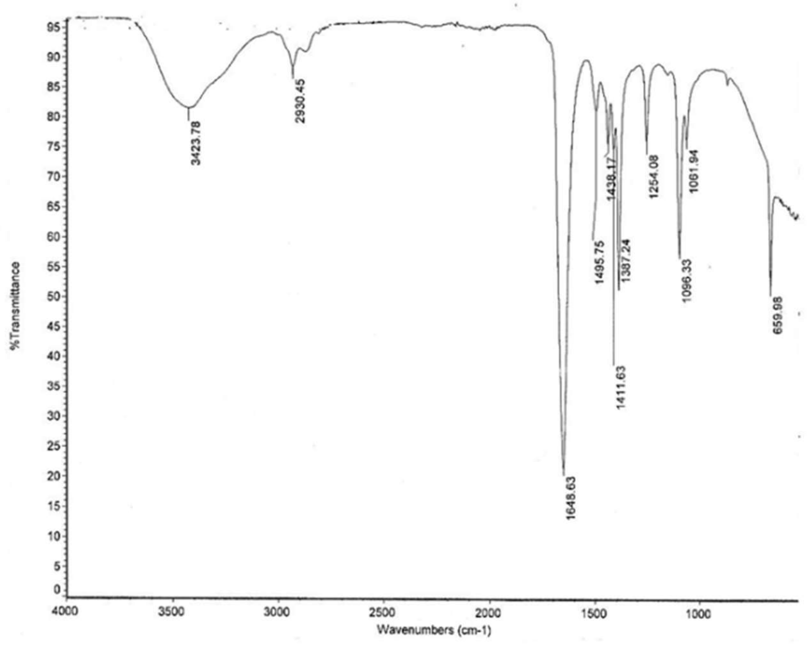

S 10. FT-IR spectrum of methanol fraction of *A. americana.*

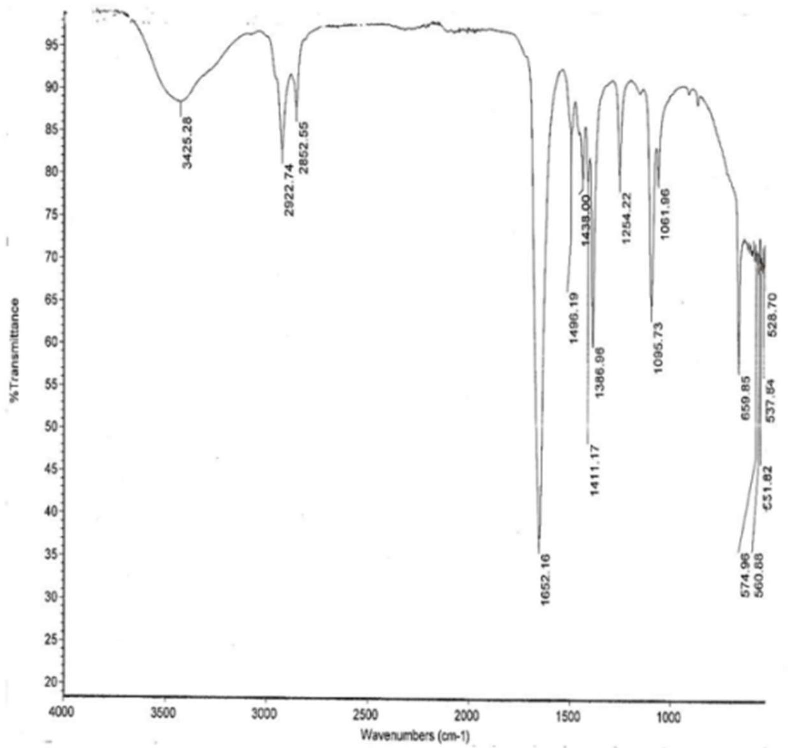

S 11. FT-IR spectrum of n-Hexane fraction of *A. americana.*
